# Lung Cancer Screening: Are You Ready?

**Published:** 2014-05-01

**Authors:** Wendy H. Vogel

**Affiliations:** Ms. Vogel is a nurse practitioner at Wellmont Cancer Institute in Kingsport, Tennessee.

Screening for lung cancer is now recommended for certain people at high risk for the development of this malignancy. Currently, there is no evidence that other risk groups should be screened.

## Risk Factors and Smoking Cessation

The National Comprehensive Cancer Network (NCCN) Guidelines define persons at high risk of lung cancer as those who have more than a 30–pack-year smoking history, who are between 55 and 74 years of age, and who are current smokers or have stopped smoking within the past 15 years (NCCN, 2014). Table 1 defines the different risk categories. Additional risk factors that could raise the risk status are also included, such as radon or other occupational exposures.

**Table 1:**
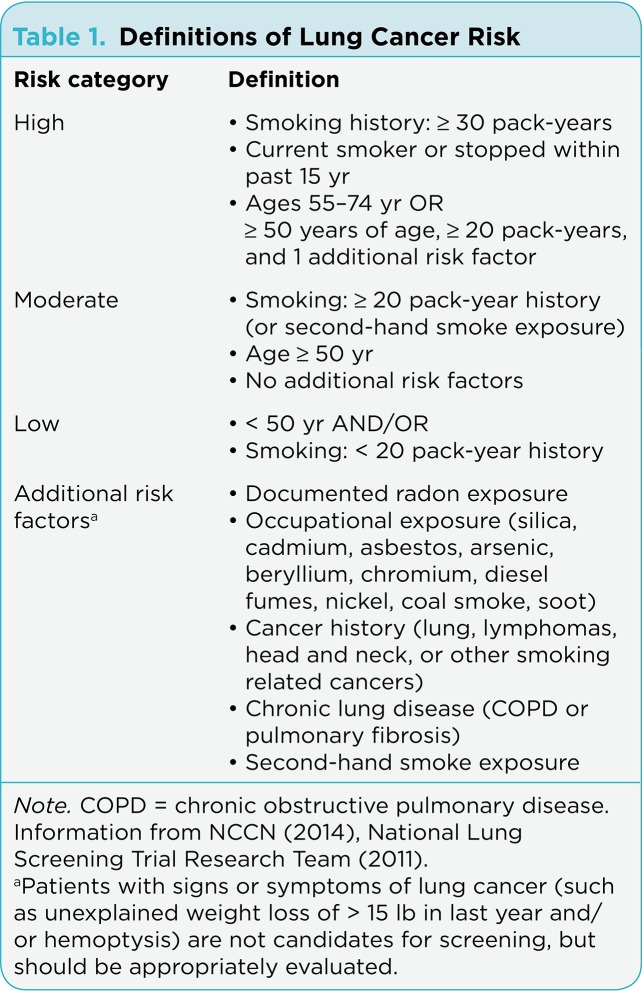
Definitions of Lung Cancer Risk

In 2011, the National Lung Screening Trial (NLST) estimated that there were about 7 million people in the United States who would meet high-risk eligibility criteria for lung cancer screening. However, an estimated 45.3 million people in the United States (19.3% of all adults) are current smokers (Centers for Disease Control and Prevention [CDC], 2013a). About 34.6% of adults with less than a high school education are current smokers, and 13.2% of adults with a college degree are current smokers (CDC, 2013b).

Tobacco use, which continues to be the single greatest risk factor for lung cancer, is associated with a 20-fold increase in the risk of developing lung cancer. Lung cancer screening must never replace or be offered without smoking cessation education. There is concern that lung cancer screening will be used as an excuse for patients to continue smoking (Wender et al., 2013), as those who receive a negative result from screening may have a false sense of security. Gomez and LoBiondo-Wood (2013) summarized the current literature on the effects of lung cancer screening on smoking cessation rates. They concluded that trial participants (both in the screening and control arms) were more likely to stop smoking compared with the general population.

## Evidence-Based Screening Guidelines

Various methods for lung cancer screening have been studied, including sputum testing, chest x-ray, and computed tomography (CT), but the most effective method has been shown to be screening with low-dose CT scans.

## Older Trials

The Early Lung Cancer Action Project (ELCAP) found that low-dose CT can improve the detection of early lung cancer (Henschke et al., 1999). In 2006, results of a large collaborative lung cancer screening study were published in *The New England Journal of Medicine* (International Early Lung Cancer Action Program Investigators, 2006). This study followed over 30,000 asymptomatic persons at risk for lung cancer who underwent low-dose CT scanning. The investigators concluded that annual spiral CT screening can detect curable lung cancers. Prior to this study, a significant concern was whether such screening and early intervention were sufficiently effective to justify screening large asymptomatic at-risk populations. This study found that lung cancer detection rates were similar to or slightly higher than rates of detection in the breast cancer screening setting. Cost-effectiveness was also noted to be positive and similar to that associated with mammography screening.

Another study found that although low-dose CT screening might increase the rate of lung cancer detection, it might not reduce the risk of advanced lung cancer or mortality from lung cancer (Bach et al., 2007).

## National Lung Screening Trial

New guidelines recommending lung cancer screening in certain high-risk populations are based on data derived from the NLST (Detterbeck, Mazzone, Naidich, & Bach, 2013; Gomez & LoBiondo-Wood, 2013; NCCN, 2014; Wender et al., 2013). Table 2 summarizes the population criteria recommended for lung cancer screening.

**Table 2:**
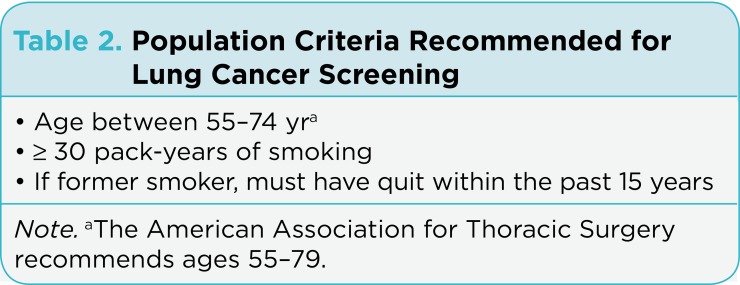
Population Criteria Recommended for Lung Cancer Screening

These factors also defined the criteria for entrance into the NLST (NLST Research Team, 2011), funded by the National Cancer Institute. Individuals with a previous diagnosis of lung cancer, previous CT scanning within 18 months, hemoptysis, or unexplained weight loss were excluded. Over 53,000 people at high risk for lung cancer were enrolled across 33 US medical centers and randomly assigned to receive three annual screenings with either low-dose CT or single-view posteroanterior chest radiography. Data showed that low-dose CT screening reduced mortality from lung cancer by 20%.

## A Comprehensive, Coordinated Care Process

Counseling about the risks and benefits of lung cancer screening prior to actual screening is recommended (Detterbeck et al., 2013). Issues to be discussed during counseling are described in Table 3. Knowing the risks and benefits of lung cancer screening will enable patients to make more informed decisions. It is difficult to quantify some risk, such as psychological concerns or the impact of radiation exposure. Overdiagnosis is also a risk: The identification of a small, indeterminable, likely benign nodule (too small to biopsy) may lead to unnecessary worry and an invasive procedure for something that might never become symptomatic during a patient’s lifetime (Boiselle, 2013). Perceptions of risk and fears of cancer are widely varied, highlighting the importance of individual decision-making with appropriate education (Park et al., 2013).

**Table 3:**
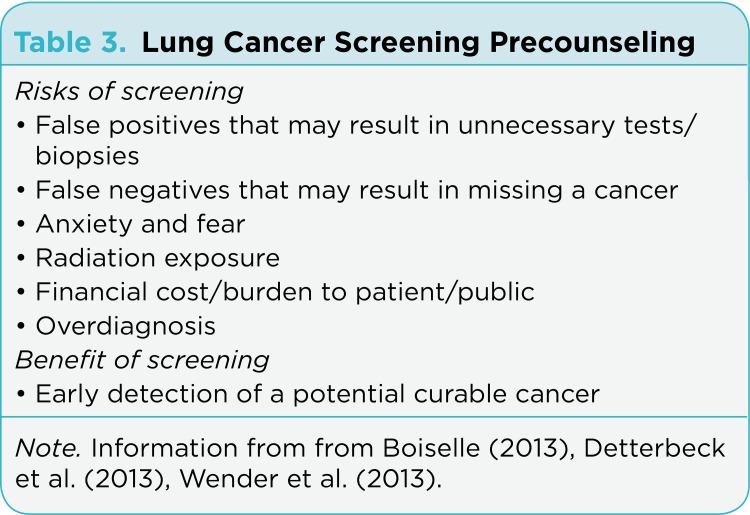
Lung Cancer Screening Precounseling

Lung cancer screening should be conducted in a multidisciplinary setting (Detterbeck et al., 2013; Wender et al., 2013). This would include certified radiologists and radiologic technologists with additional training/expertise in screening image quality and interpretation (NLST Research Team, 2011). Quality metrics similar to those used for mammography must be developed. A comprehensive, coordinated care process is required for patient selection, patient counseling, smoking cessation intervention, screening, image interpretation, management of findings (including referral to multidisciplinary team if indicated), and data collection. A formal procedure for the evaluation of any lung nodules must be defined as predetermined algorithms. This step will minimize unnecessary imaging and biopsies (Detterbeck et al., 2013). At this time, the duration of screening is unknown; the NLST performed three annual screenings (NLST Research Team, 2011).

## The Role of the Advanced Practitioner

The advanced practitioner (AP) in oncology has an important role in screening for lung cancer. It is unknown whether primary care practitioners will be able to assume a major role in this process (Detterbeck et al., 2013). Education of members of the public, professionals, and insurers is vital to the screening process. Resources for the AP, including educational materials and information on tobacco cessation, are listed in the upper section of Table 4. The lower section of Table 4 offers some resources that may assist in patients’ understanding of the screening process and its implications.

**Table 4:**
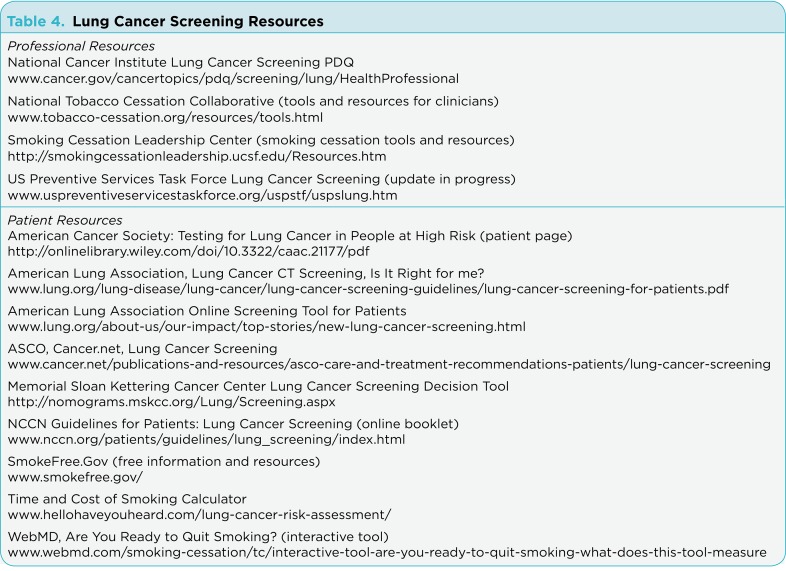
Lung Cancer Screening Resources

Advanced practitioners may be consulted regarding who is at risk and who qualifies for screening. Counseling about potential benefits and potential harms is within the scope of the AP as well. Smoking cessation counseling may also be performed by the oncology AP. The AP may be influential in obtaining insurance coverage for lung cancer screening. Further research is also needed to define the screening model and to answer questions regarding the duration of screening, cost-effectiveness, and effect upon lung cancer mortality.
